# Lactomycins A–C, Dephosphorylated Phoslactomycin Derivatives That Inhibit Cathepsin B, from the Marine-Derived *Streptomyces* sp. ACT232

**DOI:** 10.3390/md16020070

**Published:** 2018-02-21

**Authors:** Yi Sun, Rogie Royce Carandang, Yuta Harada, Shigeru Okada, Kazutoshi Yoshitake, Shuichi Asakawa, Yuichi Nogi, Shigeki Matsunaga, Kentaro Takada

**Affiliations:** 1 Laboratory of Aquatic Natural Products Chemistry, Graduate School of Agricultural and Life Sciences, The University of Tokyo, Tokyo 113-8657, Japan; sylotus@hotmail.com (Y.S.); rrzc_8@yahoo.com (R.R.C.); y_harada0609@yahoo.co.jp (Y.H.); aokada@mail.ecc.u-tokyo.ac.jp (S.O.); 2 Laboratory of Aquatic Molecular Biology and Biotechnology, Graduate School of Agricultural and Life Sciences, The University of Tokyo, Tokyo 113-8657; akyoshita@g.ecc.u-tokyo.ac.jp (K.Y.); asakawa@mail.ecc.u-tokyo.ac.jp (S.A.); 3 Japan Agency for Marine-Earth Science and Technology (JAMSTEC), Natsushima, Yokosuka, Kanagawa 237-0061, Japan; nogiy@jamstec.go.jp

**Keywords:** *Streptomyces*, cathepsin B, enzyme inhibitory, phoslactomycin

## Abstract

Three new polyketides, lactomycins A (**1**)–C (**3**), were isolated from the culture broth of a marine-derived *Streptomyces* sp. ACT232 as cathepsin B inhibitors. Their structures were determined by a combination of NMR and MS data analyses to be the dephosphorylated derivatives of a phoslactomycin class of metabolites. Lactomycins exhibited cathepsin B inhibitory activity (IC_50_ 0.8 to 4.5 μg/mL). Even though the biosynthetic gene clusters found in the genome of the current strain have high similarity to those of phoslactomycin, neither phoslactomycins nor leustroducsins were detected by LC-MS analyses of the crude extract.

## 1. Introduction

The majority of the structurally and functionally diverse anticancer agents have been derived from terrestrial naturel products [1]. Although marine organisms were reported to exhibit a high incidence of cytotoxic activity [2], it is only recently that a few anticancer agents derived from marine organisms have been approved [3]. We have been searching for anticancer compounds from marine organisms [4], and payed attention to cathepsin B as a target to discover anticancer agents with a mode of action distinct from cell cycle arrest. Cathepsin B is a lysosomal protease of the papain family, which has been considered as a superb target for cancer chemotherapy [5]. It is activated and secreted in the tumor microenvironment and enhances tumor metastasis and infiltration by cleaving extracellular matrix proteins and activating several prominent proteases. It is overexpressed in various cancers and its knockout retards cell proliferation and tumor growth. During our screening program it was found that the crude extract of the *Streptomyces* sp. ACT232, a marine-derived actinomycete, showed cathepsin B inhibitory activity [6]. We isolated and studied the structures and biosynthesis of the active constituents.

## 2. Results

### 2.1. Isolation

**Figure 1:**
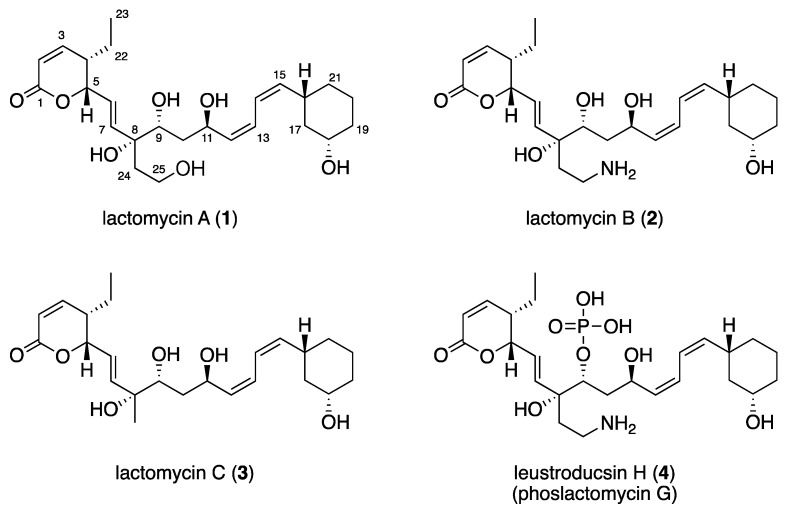
Structures of lactomycins A (**1**)–C (**3**) and phoslactomycin G (**4**).

The mycelia from the culture of *Streptomyces* sp. ACT232 were collected by vacuum filtration. Resins (Amberlite XAD 16N) were added to the filtrate to allow adsorption of the metabolites. The acetone extracts of the mycelia and resins were combined and partitioned between *n*-BuOH and H_2_O. The *n*-BuOH fraction was separated by ODS flash chromatography to give five fractions. The active fraction was separated by reversed-phase HPLC to afford three metabolites termed lactomycins A–C (Figure 1, **1**–**3**).

### 2.2. Structure Elucidation

The molecular formula of lactomycin A (**1**), C_25_H_38_O_7_, was established by HRESIMS. The analyses of ^1^H NMR and HSQC spectra of **1** indicated the presence of eight protonated sp^2^ carbons (δ_H_/δ_C_ 7.03/151.5, 6.10/122.5, 6.09/121.8, 5.96/120.5, 5.84/137.4, 5.69/123.7, 5.40/137.6, 5.25/137.8), four oxymethines (δ_H_/δ_C_ 5.02/80.7, 4.58/63.5, 3.53/72.6, 3.37/68.5), one oxygenated methylene (δ_H_/δ_C_ 3.53, 3.46/57.9), six methylenes, and one terminal methyl (Table 1). The ^13^C NMR spectrum revealed the presence of two additional non-protonated carbons at δ_C_ 164.0 and 76.6 (Table 1). Three partial structures of C2–C7 plus C22–C23, C9–C21, and C24–C25 were established by interpretation of the ^1^H-^1^H COSY spectrum. HMBC correlations from H2, H3, and H5 to the carbon at δ_C_ 164.0 (C1) indicated that C2 to C5 comprised an α,β-unsaturated δ-lactone moiety. There was a 1,3-disubstituted cyclohexane moiety (C16 to C21) in the second partial structure, in which C18 was substituted by an oxygen atom judging from the ^1^H and ^13^C NMR chemical shifts (δ_H_/δ_C_ 3.37/68.5). The planar structure of the C9 to C15 portion was elucidated by analysis of the COSY spectrum. The third partial structure was composed of disubstituted ethane in which a substituent at C25 was assigned as an oxygen atom on the basis of the ^1^H and ^13^C NMR data (δ_H_/δ_C_ 3.46, 3.53/57.9). The three partial structures were all attached to the tetrasubstituted carbon bearing an oxygen atom (C8), as demonstrated by HMBC correlations from H7, H9, and H_2_-24 to C8. Considering the molecular formula, the oxygen atoms at C8, C9, C11, C18, and C25 were all assigned as free hydroxyl groups.

The coupling constant of 9.7 Hz between H2 and H3 suggested that they are cis to each other, which was supported by a prominent NOESY cross peak between these protons. The *E*-geometry of the C6 to C7 double bond was assigned from the coupling constant of 15.6 Hz between H6 and H7. Methylene protons on C10 were diastereotopic and coupling between H10a (δ_H_ 0.98)/H9 (10 Hz), H10a/H11(1.4 Hz), and H10b (δ_H_ 1.58)/H11 (10 Hz) was observed, whereas coupling between H10b/H9 was not observed. Analysis of these coupling constant values suggested the 9R*, 11R* relative configuration. The coupling constants between H12 and H13 and between H14 and H15, as well as between H13 and H14, were determined by analyzing the AB-multiplet for H13 and H14, which suggested that the three coupling constants were all 11 Hz, indicating that the C12 to C13 and C14 to C15 double bonds were both *Z*. This assignment was supported by intense NOESY cross peaks H12/H13, H14/H15, H11/H14, and H13/H16. The C16 and C18 substituents of the cyclohexane ring were both equatorial, as shown by the large coupling constants observed for H16 and H18 to the adjacent axial methylene protons. The structural assignment conducted so far suggested **1** to be a dephospho-25-deamino-25-hydroxy-leustroducsin H (phoslactomycins G) (**4**) derivative [7,8]. Although the relative stereochemistry within the three partial structures was identical with that of leustroducsin H [9], due to the insulation by double bonds and the possible free rotation of the bond between C7 and C8, it was not possible to correlate the relative configurations between the partial structures nor assign that of C8 on the basis of the NOESY data. The NMR data of **1** was very close to those reported for phoslactomycins [10,11,12,13] and leustroducsins [14,15], except for those around C9 and C18, which were phosphorylated and esterified, respectively, in the latter compounds. The presence of an almost identical biosynthetic gene cluster with those previously reported for phoslactomycins (vide infra) suggests that the stereochemistry is shared between lactomycin A and phoslactomycins.

Lactomycin B (**2**) had a molecular formula of C_25_H_39_NO_6_ as determined by HRESIMS. Extensive 1D and 2D NMR analyses suggested that **2** had a carbon backbone identical to that of **1**. Chemical shift values of C25 methylene (δ_H_/δ_C_ 3.03, 2.97/37.4) in **2** were significantly different from those in **1** (δ_H_/δ_C_ 3.53, 3.46/57.9) and suggested that C25 was substituted by an amino group. Therefore, compound **2** was a dephosphorylated derivative of leustroducsin H. 

The molecular formula of lactomycin C (**3**) was determined to be C_24_H_36_O_6_, which is smaller than **1** by a CH_2_O unit. ^1^H and ^13^C data of **3** were almost identical to those of **2,** except for the replacement of the aminoethyl group by a methyl group (δ_H_/δ_C_ 1.27/24.8). This substitution was confirmed by interpretation of the 1D and 2D NMR data.

### 2.3. Biosynthetic Gene Cluster

**Figure 2:**
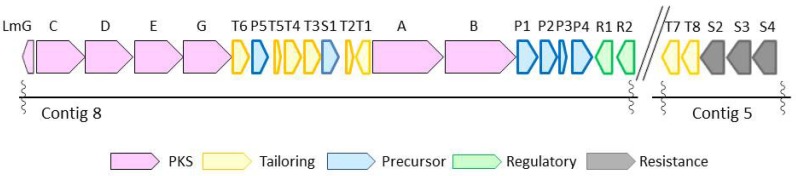
Biosynthetic gene cluster of lactomycins from *Streptomyces* sp. ACT232.

With structural difference only being exhibited in the state of phosphorylation and acylation, lactomycins can be considered as biosynthetic intermediates of phoslactomycins. Because dephospho-25-deamino-25-hydroxy phoslactomycin B (NPLM), which is closely related to lactomycin A, has been isolated from the streptomycete that produces leustroducsins [16], we suspected that our streptomycete has the same sets of biosynthetic genes to produce phoslactomycins. We set out to reveal the biosynthetic gene cluster of lactomycins by the genome analysis of *Streptomyces* sp. ACT232. The genome was sequenced using Illumina Hiseq, followed by assembly with CLC Genomics Workbench to give 15 contigs which cover 7.5 Mbp in total. The sequence data in this study have been deposited in GenBank under Genbank ID LC364194 and LC365285. Analyses of contigs by antiSMASH 3.0 [17] implied the presence of biosynthetic gene clusters of 32 secondary metabolites, including a gene cluster parallel to those reported for the biosynthetic gene cluster of phoslactomycin. Biosynthetic gene clusters of phoslactomycins were previously identified in two species of streptomycetes, namely *Streptomyces* sp. HK-803 [18,19,20] and *Streptomyces platensis* SAM-0654 [21]. Whilst they contain almost the same sets of genes, their architectures are different; the gene cluster of phoslactomycin in *Streptomyces* sp. HK-803 is present in one cluster, whereas the gene cluster in *Streptomyces platensis* SAM-0654 is dispersed into two clusters. The lactomycin cluster is composed of six genes for the biosynthesis of cyclohexanecarboxyl-CoA (CHC) as the starter unit, six PKS genes, eight genes for post PKS modifications, four transporters, and two regulatory genes (Figure 2). The biosynthetic gene cluster of our strain was separately located in two contigs, as found in *Streptomyces* sp. SAM-0654, and all genes required for the biosynthesis of phoslactomycins exist in the genome. The deduced function of each lactomycin biosynthetic gene and identity with the previously reported corresponding genes are listed in Table 2. Our genes shared identity ranging from 83% to 98% with those of the HK803 strain and identity ranging from 83% to 97% with those of the SAM-0654 strain. The analysis suggested that the strain ACT232 should also produce a phoslactomycin class of metabolites. Nevertheless, we were not able to detect these metabolites in our culture by LC-MS, implying that the putative kinase (lmT4) and acyltranferase (lmT8) [22] are not functional for some reason.

### 2.4. Biological Activity

Lactomycin A, B, and C inhibit cathepsin B with IC_50_ values of 4.5 μM, 0.8 μM, and 1.6 μM, respectively. Their inhibitory activities are moderate compared with those of known cathepsin B inhibitors, such as E-64, which are active at a nM range [23]. Additionally, the cytotoxic activity of lactomycins A–C was examined, but none of them showed activity against HeLa cells at concentrations lower than 40 μM.

## 3. Discussion

Phoslactomycins [10,11,12,13] and leustroducsins [14,15] are polyketides that possess α,β-unsaturated δ-lactone and cyclohexane moieties at both ends, which are tethered through a highly unsaturated chain with a phosphate ester. This class of compounds shows a plethora of biological activities, such as antifungal activity, inhibitory activity against protein phosphatase 2A, increasing the level of cytokines such as colony stimulating factors, and induction of myeloid differentiation in HL-60 cells. However, their inhibitory activity against cathepsin B has not been reported so far. We noticed the structural similarity of lactomycins and leptomycins [24,25], polyketides with an α,β-unsaturated δ-lactone moiety and an unsaturated chain. Leptomycins are potent cytotoxins and the active site is the α,β-unsaturated δ-lactone moiety. However, lactomycins do not show cytotoxic activity, demonstrating that the presence of α,β-unsaturated δ-lactone is not sufficient for cytotoxicity. 

## 4. Materials and Methods 

### 4.1. General Experimental Procedures

UV spectra were measured on a Shimadzu Biospec 1600 (Shimadzu, Kyoto, Japan). NMR spectra were recorded on a JEOL alpha 600 NMR spectrometer (JEOL, Tokyo, Japan). Chemical shifts were referenced to a solvent peak: δ_H_ 2.49 and δ_C_ 40.0 (DMSO-d_6_) and δ_H_ 3.30 and δ_C_ 49.0 (CD_3_OD). HRESI mass spectra were measured on a JEOL JMS-T100LC (JEOL, Tokyo, Japan). HPLC purification was carried out on a Shimadzu LC 20AT (Shimadzu, Kyoto, Japan) with an SCL-10 Avp controller and an SPD-10Avp detector.

### 4.2. Collection and Identification of the Microorganism

Deep-sea sediments were collected by the manned submersible “SHINKAI 2000” system off Hatsu-shima, Sagami-Bay, Japan, at a depth of 1174 m, in December 2001. The sediment sample was stored in a sterilized sampler, frozen with liquid nitrogen, and transported to the laboratory, where it was kept frozen until processed. The *Streptomyces* sp. ACT232 was isolated from this sample. The taxonomy of the strain was determined by 16S rRNA phylogenetic analysis using 27F and 1492R primers, and the sequence was deposited in the DNA Data Bank of Japan (DDBJ, accession no. AB968434).

### 4.3. Fermentation, Extraction, and Isolation

*Streptomyces* sp. ACT232 was cultured in 120 × 500 mL Erlenmeyer flasks, each containing 250 mL of ISP2 medium (yeast extract 1.0 g, malt extract 2.5 g, glucose 1.0 g) at 28 °C on rotary shakers at 150 rpm. After 10 days of culture, the mycelia were separated by vacuum filtration. Resins (Amberlite XAD 16N, Sigma-Aldrich, MO, USA) were added to the filtrate to allow the adsorption of metabolites. The acetone extract of both the mycelia and resins was combined and then partitioned between *n*-BuOH and H_2_O. The extract was concentrated in vacuo and separately subjected to ODS flash column chromatography eluting with 20%, 40%, 60%, 80%, and 100% (*v*/*v*) MeOH in H_2_O. The fractions that eluted with 40% and 60% MeOH were purified by ODS-HPLC (Cosmosil MSII φ 4.6 × 250 mm, Nacalai tesque, Tokyo, Japan) with gradient elution from 45% to 60% aqueous MeOH with 0.2% AcOH to afford lactomycins A (1.5 mg) and C (0.3 mg). The fraction that eluted with 80% MeOH was purified by ODS-HPLC with gradient elution from 10% to 40% aqueous MeOH with 0.5% AcOH to afford lactomycins B (1.5 mg)

Lactomycin A (**1**): white solid; [α]^20^_D_ (c 0.09, MeOH) +30; UV (MeOH) λ_max_ 230 nm (log ε 4.12); ^1^H and ^13^C NMR, see Table 1; HRESIMS *m/z* 473.2510 [M + Na]^+^ (calcd. for C_25_H_38_NaO_7_, 473.2515).

Lactomycin B (**2**): white solid; [α]^24^_D_ (c 0.06, MeOH) +32; UV (MeOH) λ_max_ 230 nm (log ε 2.92); ^1^H and ^13^C NMR, see Table 1; HRESIMS *m/z* 472.2656 [M + Na]^+^ (calcd. for C_25_H_39_N Na O6, 472.2675).

Lactomycin C (**3**): white solid; [α]^20^_D_ (c 0.02, MeOH) +1; UV (MeOH) λ_max_ 230 nm (log ε 3.80); ^1^H and ^13^C NMR, see Table 1; HRESIMS *m/z* 443.2432 [M + Na]^+^ (calcd. for C_24_H_36_NaO_6_, 443.2410).

### 4.4. Identification of the Biosynthetic Gene Cluster

*Streptomyces* sp. ACT232 was cultured in ISP2 medium for three days at 30 °C with agitation and aeration. The genomic DNA was extracted from mycelia and isolated using QIAGEN Genomic-tip 20/G. The genome of *Streptomyces* sp. ACT232 was sequenced by Ilumina Hiseq to afford the data set consisting of 53,907,834 single 100 bp reads. These reads were subjected to de novo assembly with CLC Genomics Workbench (ver8.5) to afford 15 contigs as draft genome sequences. The function of each gene was identified by antiSMASH 3.0 [17] and Blast searches. (accession number LC364194 and LC365285)

### 4.5. Cathepsin B Inhibitory Assay

A Cathepsin B inhibitory assay was performed according to a modification of the method of Hiwasa et al. [26] The enzyme (cathepsin B from bovine spleen, Sigma C6286) was stocked at 1 unit/mL in 50 mM MES pH 6.0 and 0.1% Brij-35. The enzyme solution was diluted by 100 times with the buffer before use. The mixture of 4 μL test sample solution, 100 μL of the enzyme solution, and 50 μL of 25 μM fluorescent substrate (Z-Arg-Arg-AMC, Peptide Institute, Inc.) in DMSO was incubated at 37 °C for 30 min. The fluorescence of the liberated AMC was measured with an excitation at 345 nm and emission at 440 nm.

## Figures and Tables

**Table 1:** ^1^H NMR (600 MHz) and ^13^C NMR (150 MHz) Data for **1** in DMSO-*d*_6_ and **2** and **3** in CD_3_OD.

Position	1 (DMSO-*d*_6_)			2 (CD_3_OD)			3 (CD_3_OD)	
	δ_C_, Type	δ_H_ (*J* in Hz)	HMBC	δ_C_, Type	δ_H_ (*J* in Hz)	HMBC	δ_C_, Type	δ_H_ (*J* in Hz)
1	164.0, C			166.4, C			166.4, C	
2	120.5, CH	5.96, d (9.7)	1, 4	121.1, CH	6.03, d (9.8)	1, 4	120.8, CH	6.02, d (9.8)
3	151.5, CH	7.03, dd (4.7, 9.7)	1, 4, 5	152.8, CH	7.10, dd (5.0, 9.8)	1, 5	152.6, CH	7.10, dd (4.7, 9.8)
4	39.1, CH	2.48, m		40.6, CH	2.56, m		40.5, CH	2.53, m
5	80.7, CH	5.02, dd (6.5, 4.2)	1, 3, 6, 7, 22	82.4, CH	5.10, t (4.7)		82.2, CH	5.06 dd (4.3, 6.7)
6	123.7, CH	5.69, dd (6.5, 15.6)	5, 8	127.4, CH	5.97, m^a^	5, 7, 8	124.5, CH	5.87, dd (6.7, 15.7)
7	137.4, CH	5.84, d (15.6)	5, 6, 8	136.9, CH	5.97, m^a^	5, 6, 8	139.4, CH	6.00, d (15.7)
8	76.6, C			77.9, C			75.6, C	
9	72.6, CH	3.53, d (10.0)	7, 8, 10, 11, 24	74.7, CH	3.72, brd (9.6)	8	74.9, CH	3.68, d (9.7)
10a	39.6, CH_2_	0.98, m	9	40.5, CH_2_	1.34, m	11	40.1, CH_2_	1.31, m
10b		1.58, ddd(1.4, 10.0, 12.8)	11		1.75, m	9		1.73, m
11	63.5, CH	4.58, ddd (1.8, 8.4, 10.0)	9, 10, 13	65.5, CH	4.80^b^		65.0, CH	4.80, brt (10)
12	137.6, CH	5.40, brt (9)	10, 14	135.8, CH	5.45, brt (9)	14	135.7, CH	5.46, brt (9)
13	121.8, CH	6.09^b^	11, 15	124.4, CH	6.27, t (11)	11	123.8, CH	6.26, t (11)
14	122.5, CH	6.10^b^	12, 16	123.0, CH	6.23, t (11)	16	122.9, CH	6.23, t (11)
15	137.8, CH	5.25, brt (9)	13, 17, 21	139.3, CH	5.32, brt (9)	13	138.7, CH	5.31, brt (9)
16	35.0, CH	2.45, m		36.4, CH	2.55, m		36.1, CH	2.55, m
17a	42.7, CH_2_	0.98, m		43.0, CH_2_	1.02, m		42.8, CH_2_	1.03, m
17b		1.68, m			1.82, m	16, 18		1.84, m
18	68.5, CH	3.37, m		70.8, CH	3.54, m		70.3, CH	3.53, m
19a	35.6, CH_2_	0.99, m		36.1, CH_2_	1.13, m		35.6, CH_2_	1.13, m
19b		1.78, m			1.92, m			1.92, m
20a	23.9, CH_2_	1.26, m		25.1, CH_2_	1.37, m		24.8, CH_2_	1.38, m
20b		1.64, m			1.78, m			1.78, m
21a	32.4, CH_2_	0.89, m		33.3, CH_2_	0.99, m		33.0, CH_2_	0.98, m
21b		1.44, m			1.55, m			1.56, m
22a	21.7, CH_2_	1.32, m	3, 4, 5, 23	22.8, CH_2_	1.47, m	3, 4, 5, 23	22.5, CH_2_	1.46, m
22b		1.47, m	3, 4, 5, 23		1.65, m	3, 4, 23		1.65, m
23	11.3, CH_3_	0.80, d (7.4)	4, 22	11.4, CH_3_	0.96, t (7.4)	4, 22	11.1, CH_3_	0.96, d (7.4)
24	40.0, CH_2_	1.74, m	7, 8, 9, 25	35.2, CH_2_	1.98, m	7, 8, 25	24.8, CH_3_	1.27, s
25a	57.9, CH_2_	3.46, m	8, 24	37.4, CH_2_	2.97, m	24		
25b		3.53, m	8, 24		3.03, m	8, 24		

^a^ overlapped to each other; ^b^ burried under HDO peak; ^c^ AB part of an ABX multiplet, *J*_AB_ = 11 Hz.

**Table 2:** Proposed funtion of ORFs in lactomycin biosynthetic gene cluster from *Streptomyces* sp. ACT232.

Gene	Protein Homologues, Identity (%)		Proposed Function
	*S.plantensis* SAM-0654	*Streptomyces* sp. HK803	
lmG	pnG, 87	plm8, 83	Thioesterase
lmC	pnC, 90	plm4, 88	PKS
lmD	pnD, 90	plm5, 92	PKS
lmE	pnE, 91	plm6, 91	PKS
lmF	pnF, 92	plm7, 93	PKS
lmT6	pnT6, 88	plmT8, 88	Dehydrogenase
lmP5	pnP6, 94	plmT7, 94	Crotonyl CoA reductase
lmT5	pnT5, 92	plmT6, 96	Ferredoxin
lmT4	pnT4, 94	plmT5, 98	Protein kinase
lmT3	pnT3, 97	plmT4, 98	Cytochrome P450
lmS1	pnS1, 94	plmT3, 92	Transporter
lmT2	pnT2, 93	plmT2, 92	NAD dependent epimerase
lmT1	pnT1, 83	plmT1, 84	Aminotransferase
lmA	pnA, 90	plm1, 90	PKS
lmB	pnB, 90	plm2-3, 94	PKS
lmP1	pnP1, 92	plmJK, 96	AMP-dependent synthetase and ligase
lmP2	pnP2, 94	plmL, 96	Short-chain-CoA dehydrogenase
lmP3	pnP3, 93	ChcA, 97	Cyclohexenylcarbonyl-CoA reductase
lmP4	pnP4, 87	plmM, 92	NADH:FMN oxidoreductase
lmR1	pnR1, 86	plmR1, 94	Regulator
lmR2	pnR2 ^a^	plmR2, 92	Regulator
lmT7	pnT7, 92	plmS2, 90	Cytochrome P450
lmT8	pnT8, 90	plmS3, 90	18-*O*-Acyltransferase
lmS2	pnS2, 88	plmS4, 85	Transporter
lmS3	PnS3, 91	ND ^b^	ABC transporter
lmS4	PnS4, 88	ND	ABC transporter

^a^ pnR2 is not homologuos to either PImR2 or ImR2; ^b^ ND: not detected in the database.

## Supplementary Materials

The following are available online at http://www.mdpi.com/1660-3397/16/2/70/s1, 1D and 2D NMR spectra of lactomycins A (**1**)–C (**3**).
