# Supplementary material for: Lactomycins A–C, Dephosphorylated Phoslactomycin Derivatives That Inhibit Cathepsin B, from the Marine-Derived Streptomyces sp. ACT232
Source: Mar Drugs. 2018 Feb 21;16(2):70. doi: 10.3390/md16020070 (PMC5852498; doi:10.3390/md16020070)
Supplement: Supplementary file 1 [file marinedrugs-16-00070-s001.pdf]

Lactomycins A-C, Dephosphorylated Phoslactomycin Derivatives that Inhibit Cathepsin B, from the Marine-derived *Streptomyces* sp. ACT232

Yi Sun,[1,†] Rogie Royce Carandang,[1] Yuta Harada,[1] Shigeru Okada,[1] Kazutoshi Yoshitake,[2] Shuichi Asakawa,[2] Yuichi Nogi,[3] Shigeki Matsunaga,[1,*] Kentaro Takada [1,*]

[1] Laboratory of Aquatic Natural Products Chemistry, Graduate School of Agricultural and Life Sciences, The University of Tokyo, Tokyo 113-8657, Japan

[2] Laboratory of Aquatic Molecular Biology and Biotechnology, Graduate School of Agricultural and Life Sciences, The University of Tokyo, Tokyo 113-8657, Japan

[3] Japan Agency for Marine-Earth Science and Technology (JAMSTEC), Natsushima, Yokosuka, Kanagawa 237-0061, Japan

Supplementary materials

Figure S1. ^{1}H NMR spectrum of lactomycin A (**1**) in DMSO-d_6.

Figure S2. ^{13}C NMR spectrum of lactomycin A (**1**) in DMSO-d_6.

Figure S3. COSY spectrum of lactomycin A (**1**) in DMSO-d_6.

Figure S4. TOCSY spectrum of lactomycin A (**1**) in DMSO-d_6.

Figure S5. HSQC spectrum of lactomycin A (**1**) in DMSO-d_6.

Figure S6. HMBC spectrum of lactomycin A (**1**) in DMSO-d_6.

Figure S7. ^{1}H NMR spectrum of lactomycin B (**2**) in CD_3OD.

Figure S8. ^{13}C NMR spectrum of lactomycin B (**2**) in CD_3OD.

Figure S9. COSY spectrum of lactomycin B (**2**) in CD_3OD.

Figure S10. HSQC spectrum of lactomycin B (**2**) in CD_3OD.

Figure S11. HMBC spectrum of lactomycin B (**2**) in CD_3OD.

Figure S12. ^{1}H NMR spectrum of lactomycin C (**3**) in CD_3OD.

Figure S13. ^{13}C NMR spectrum of lactomycin C (**3**) in CD_3OD.

Figure S14. COSY spectrum of lactomycin C (**3**) in CD_3OD.

Figure S15. HSQC spectrum of lactomycin C (**3**) in CD_3OD.

Figure S16. HMBC spectrum of lactomycin C (**3**) in CD_3OD.

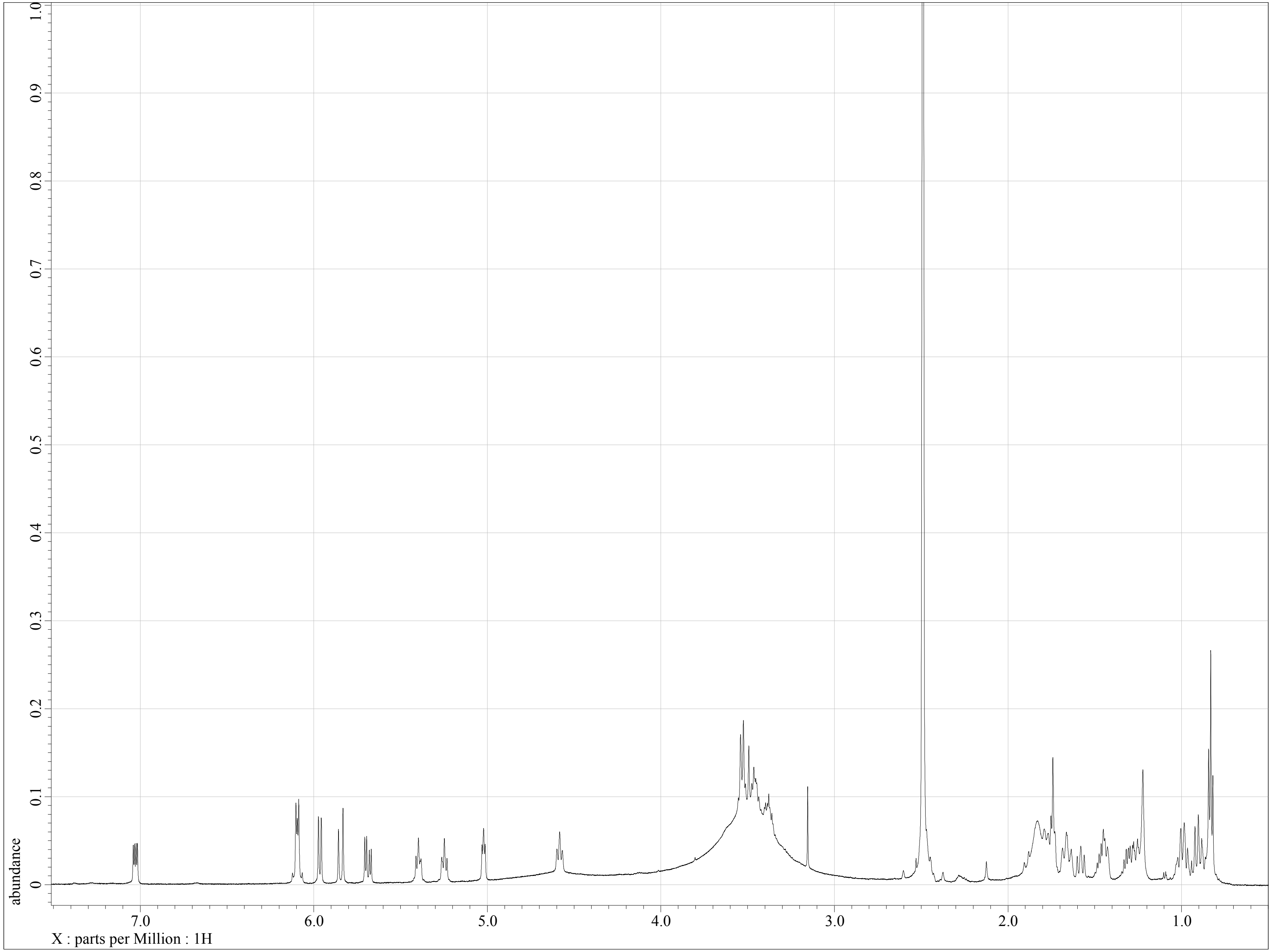

Figure S1. ^{1}H NMR spectrum of lactomycin A (**1**) in DMSO-d_6.

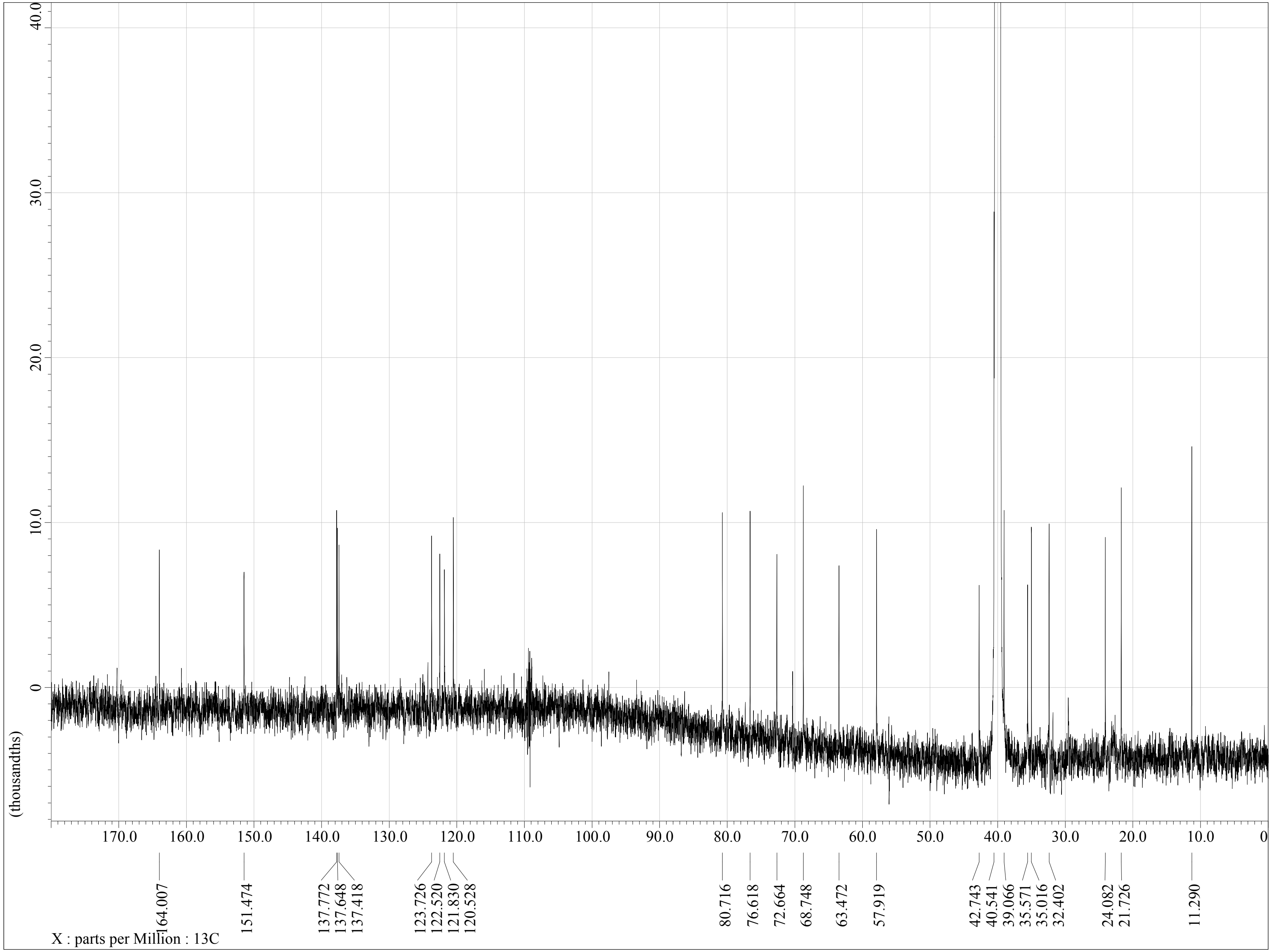

Figure S2. ^{13}C NMR spectrum of lactomycin A (**1**) in DMSO-d_6.

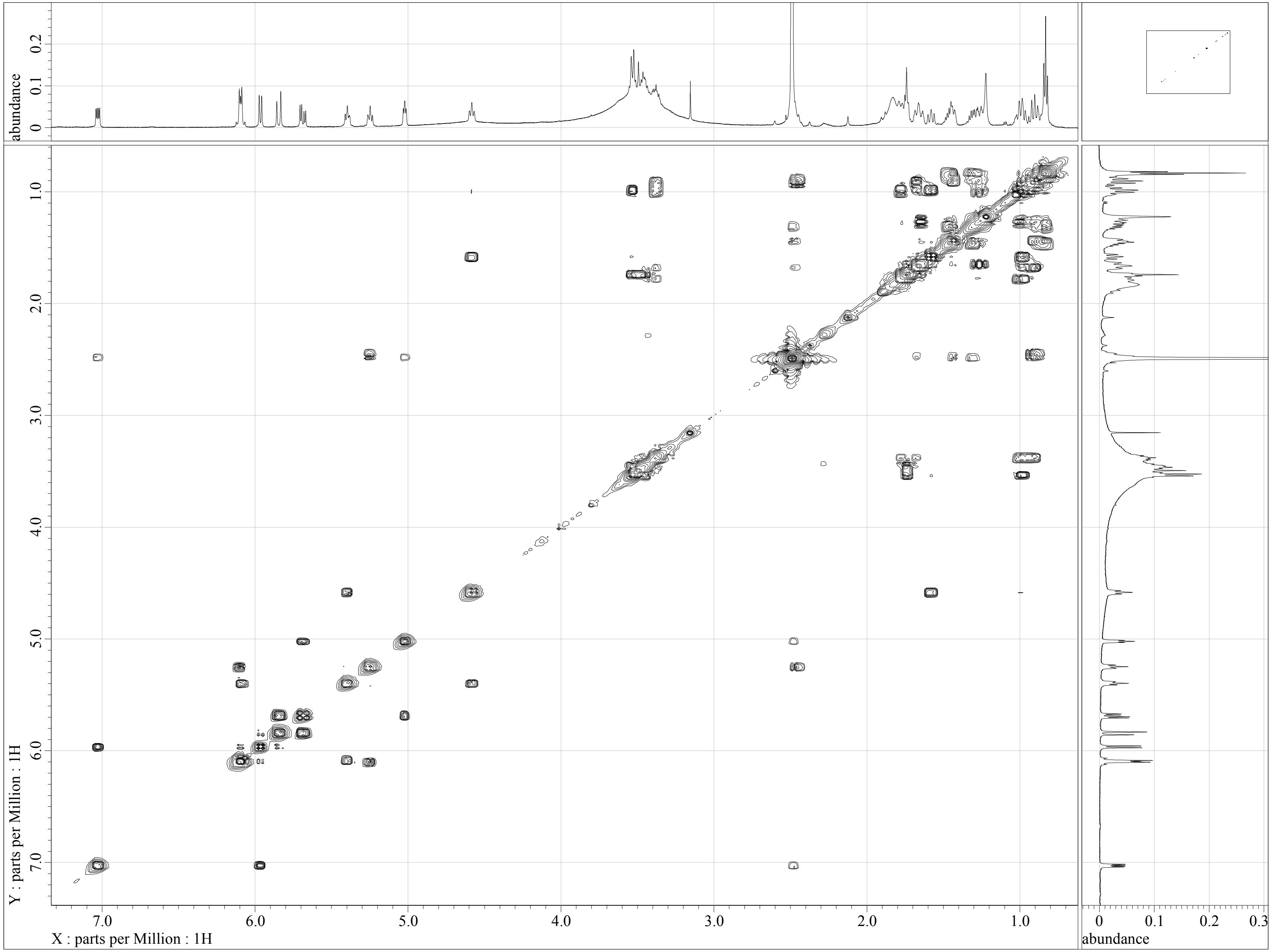

Figure S3. COSY spectrum of lactomycin A (**1**) in DMSO-*d*$_6$.

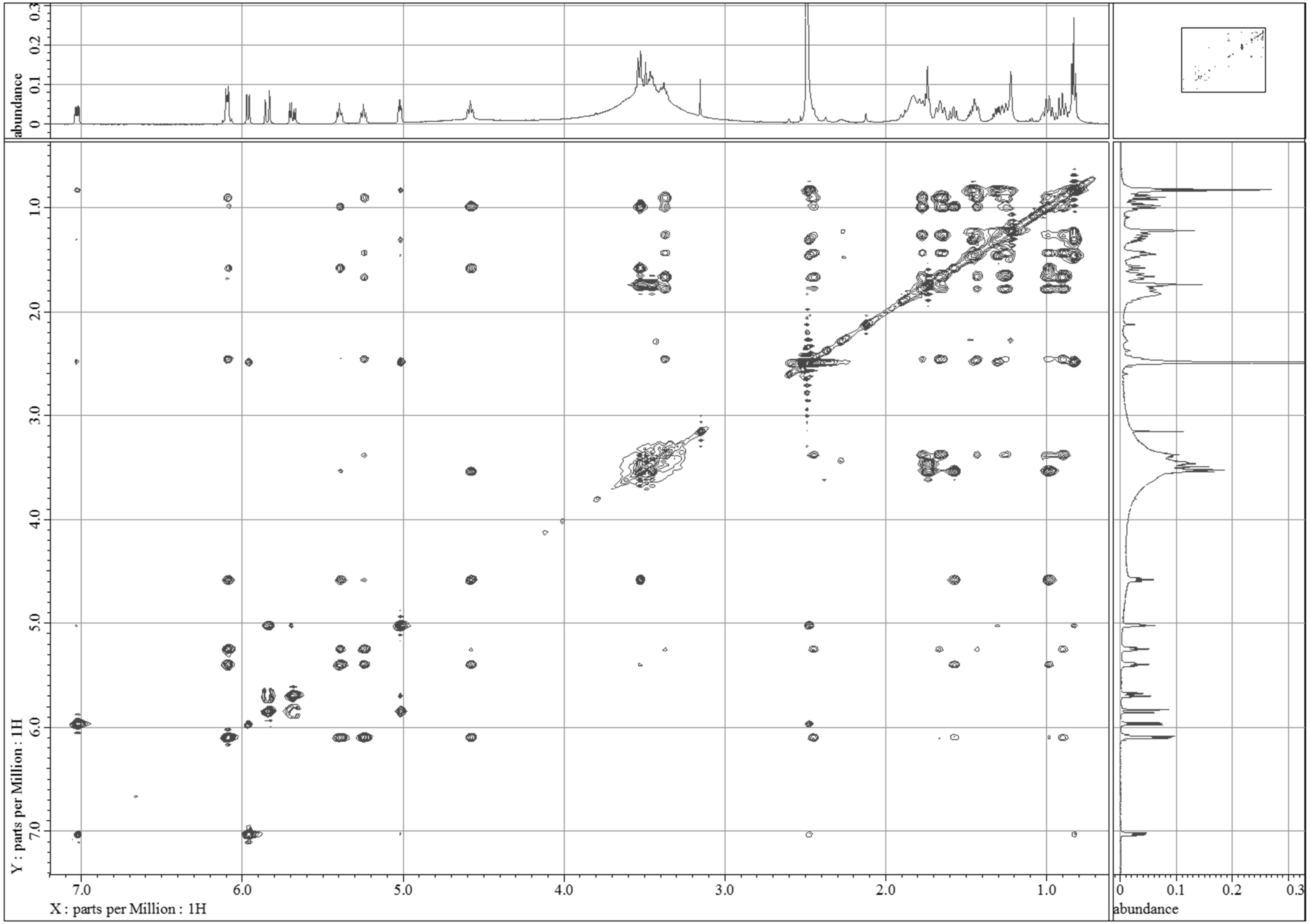

Figure S4. TOCSY spectrum of lactomycin A (**1**) in DMSO-d_6.

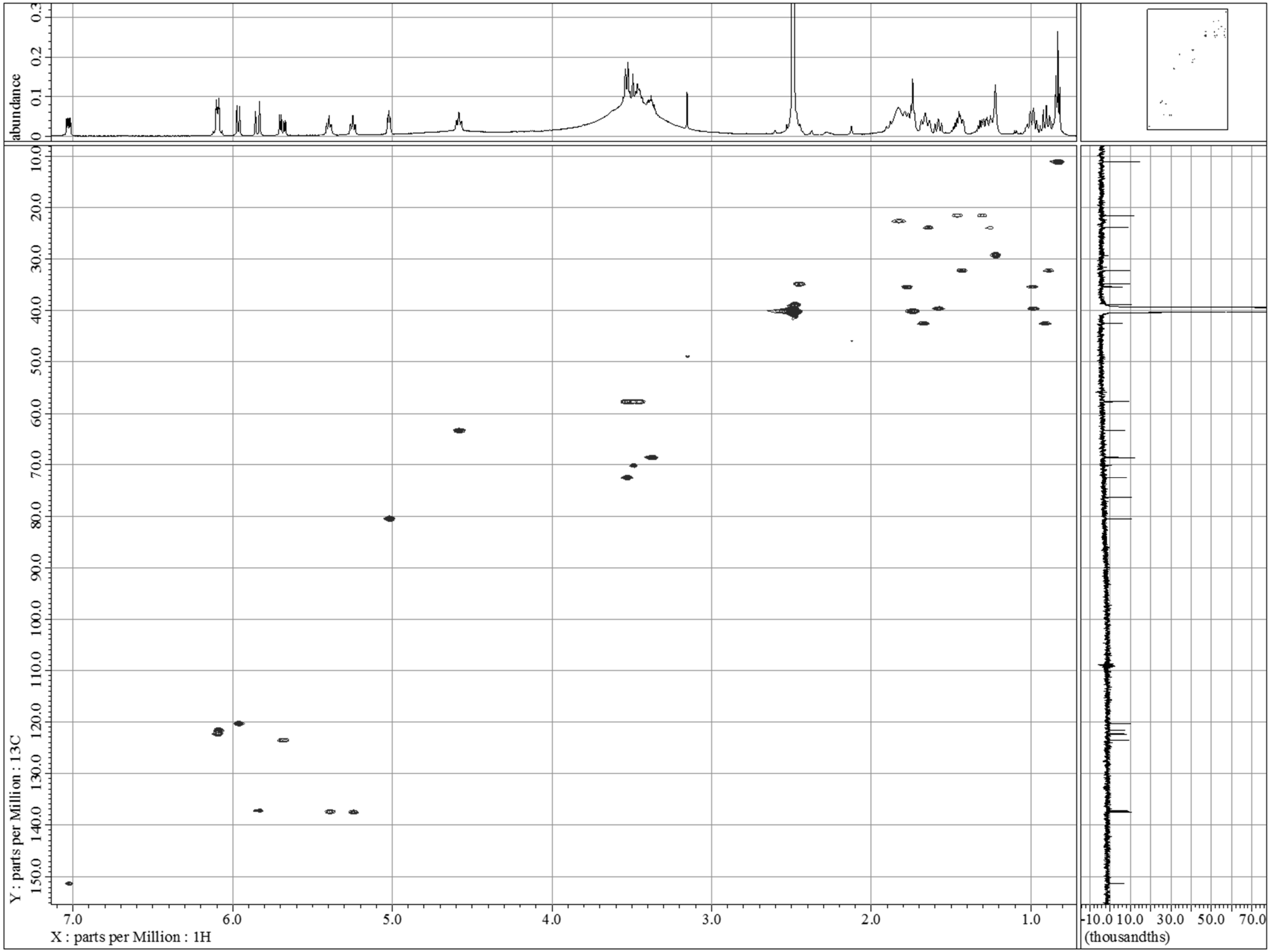

Figure S5. HSQC spectrum of lactomycin A (**1**) in DMSO-d_6

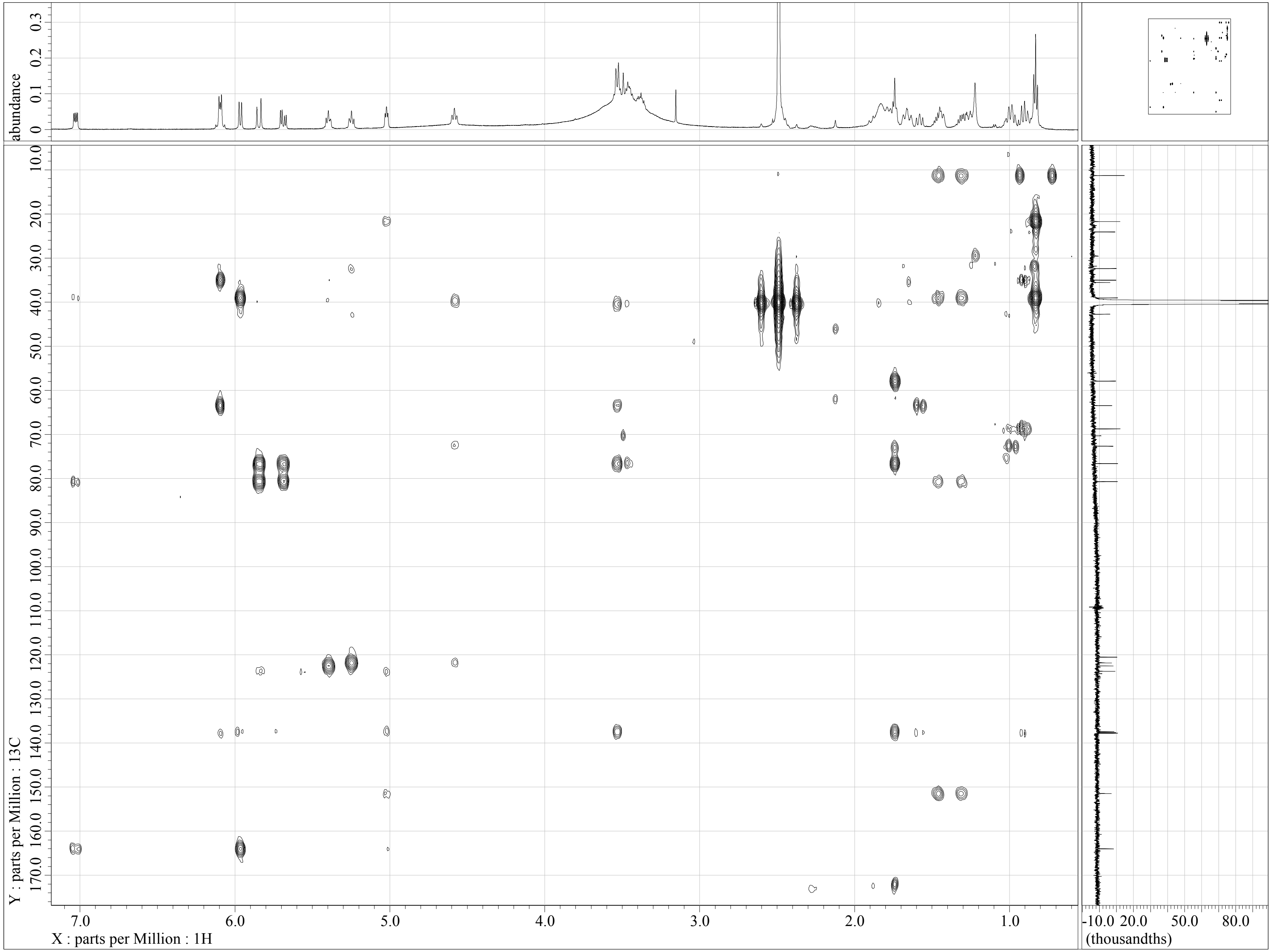

Figure S6. HMBC spectrum of lactomycin A (**1**) in DMSO-d_6

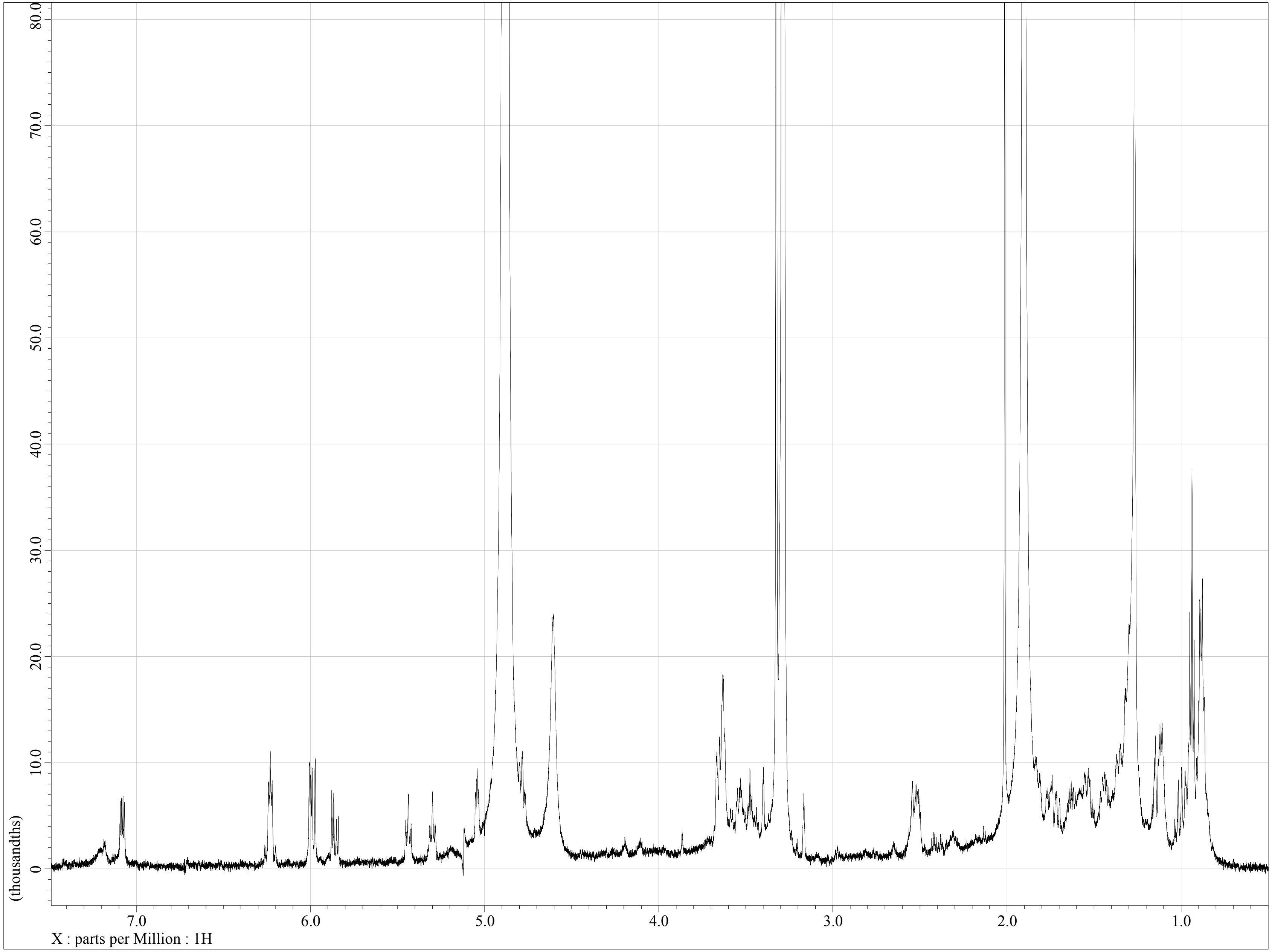

Figure S7. ^{1}H NMR spectrum of lactomycin B (**2**) in CD_3OD.

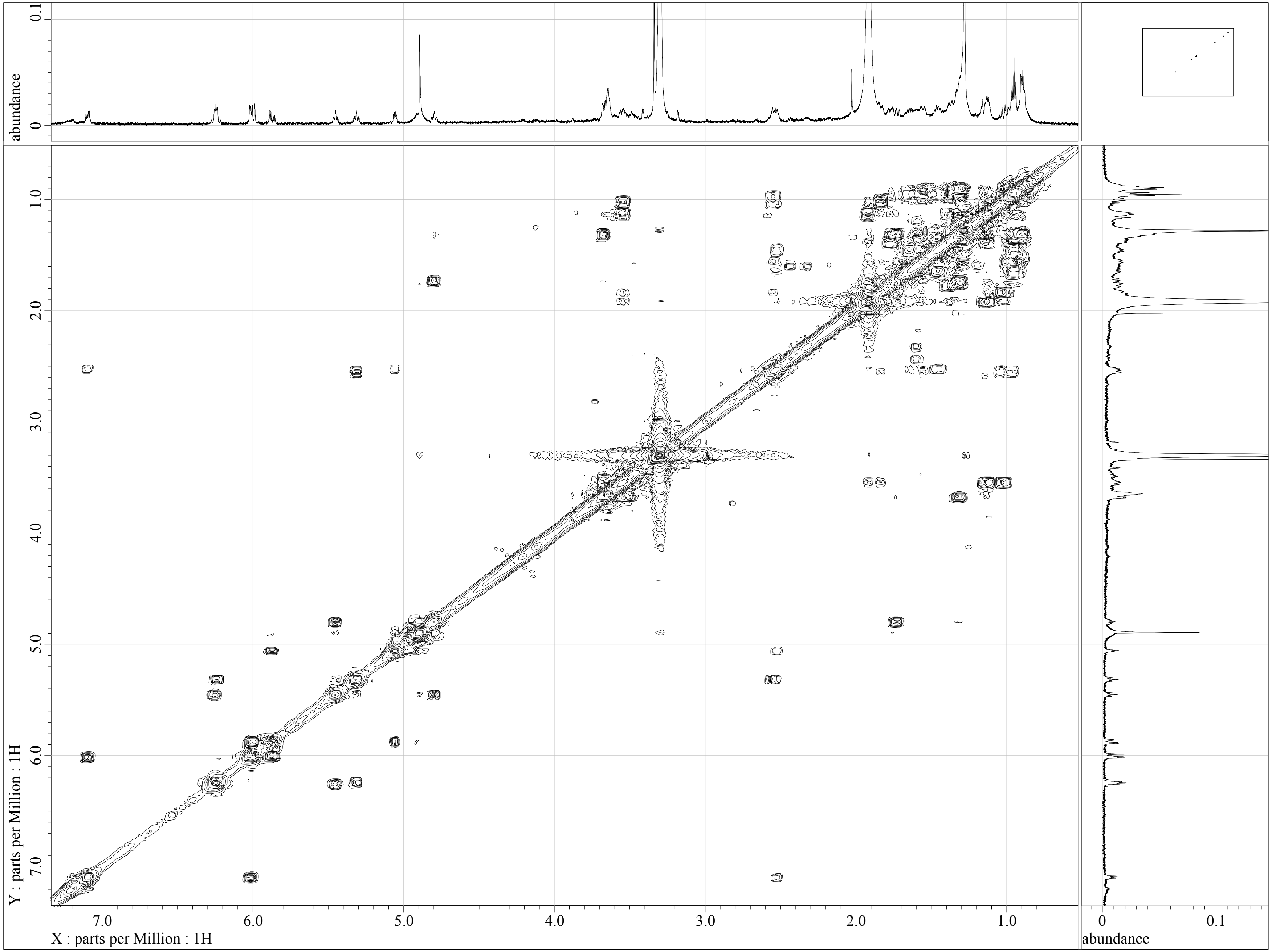

Figure S8. COSY spectrum of lactomycin B (**2**) in CD_3OD.

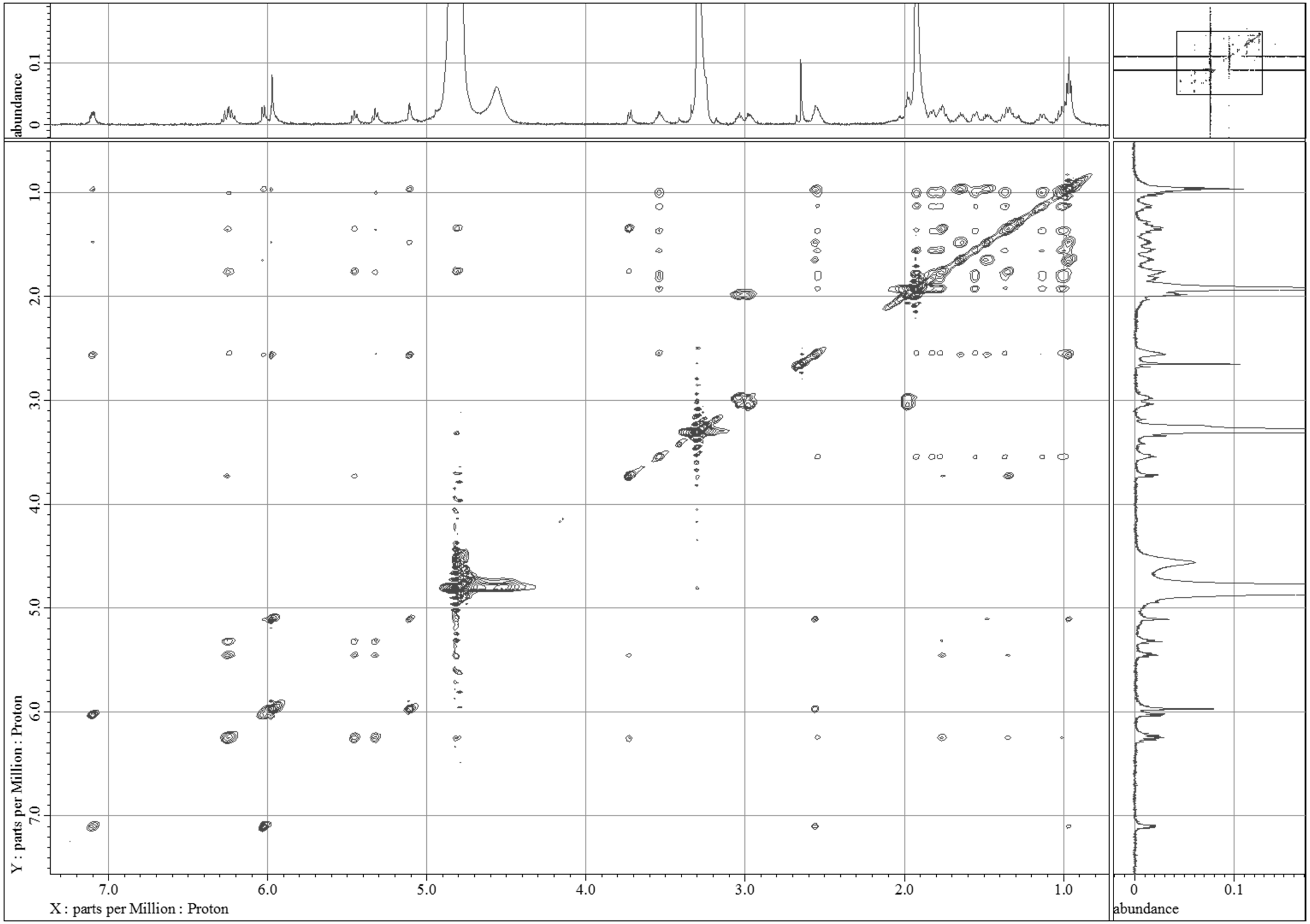

Figure S9. TOCSY spectrum of lactomycin B (**2**) in CD_3OD.

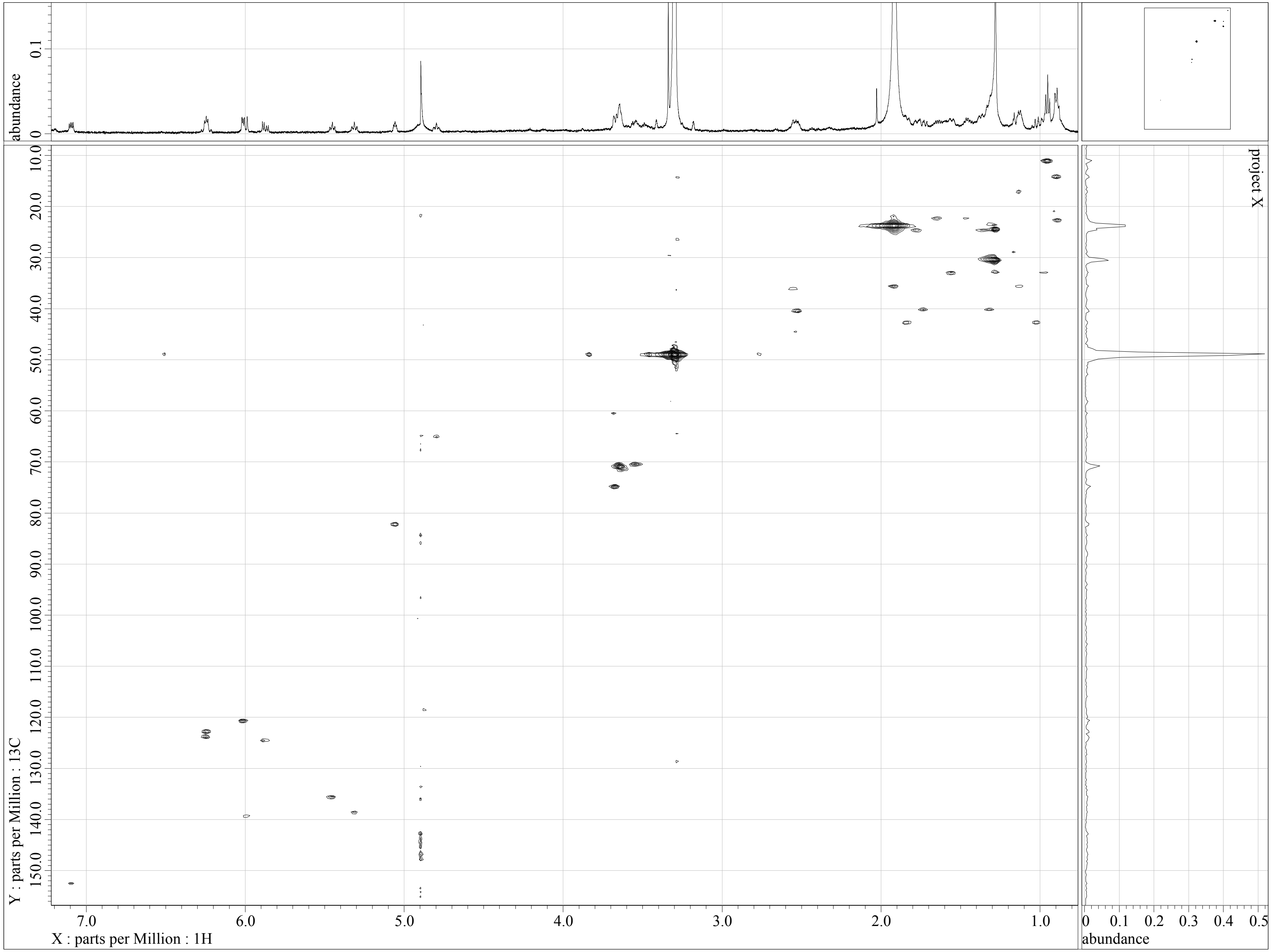

Figure S10. HSQC spectrum of lactomycin B (**2**) in CD_3OD.

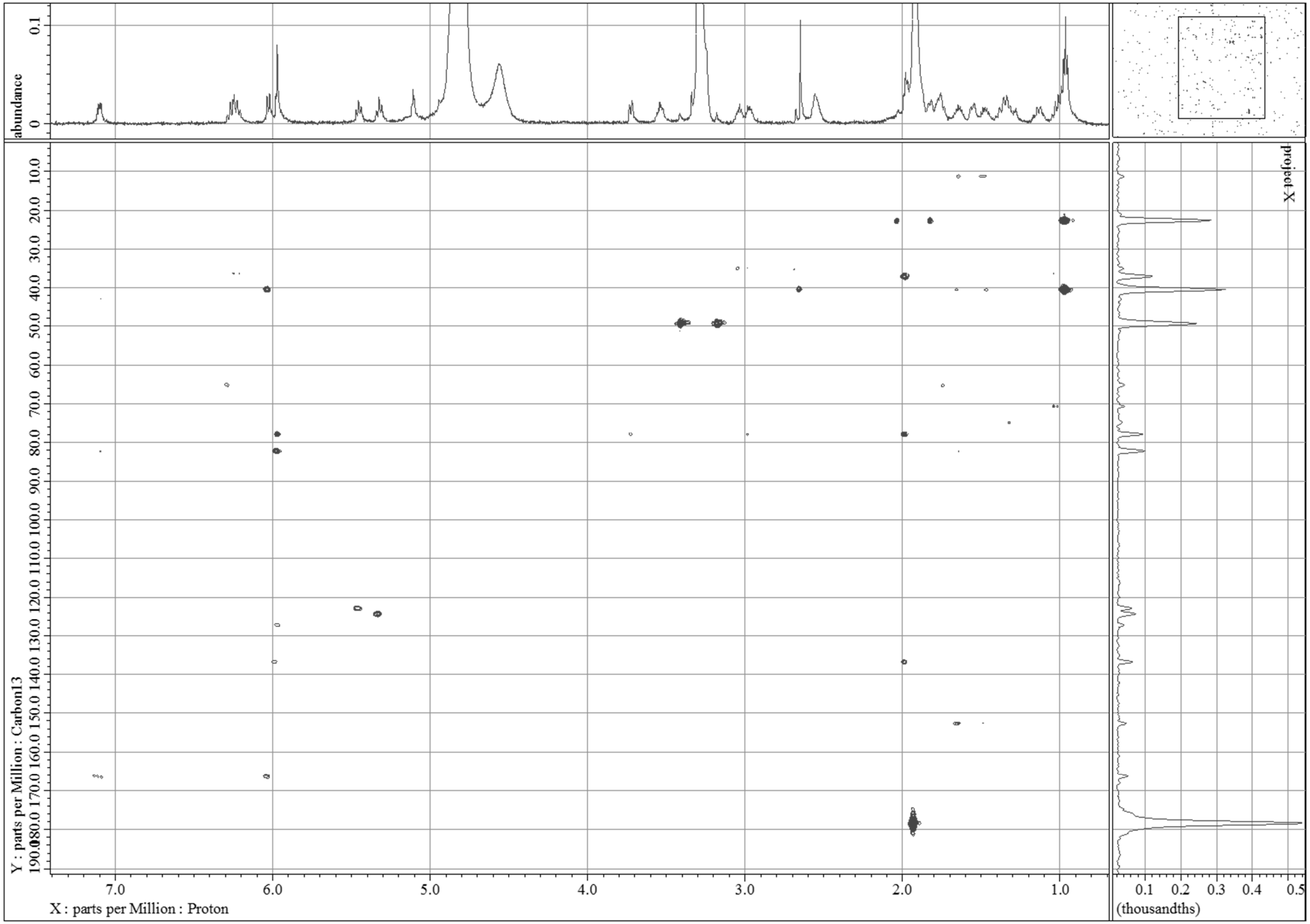

Figure S11. HMBC spectrum of lactomycin B (**2**) in CD_3OD.

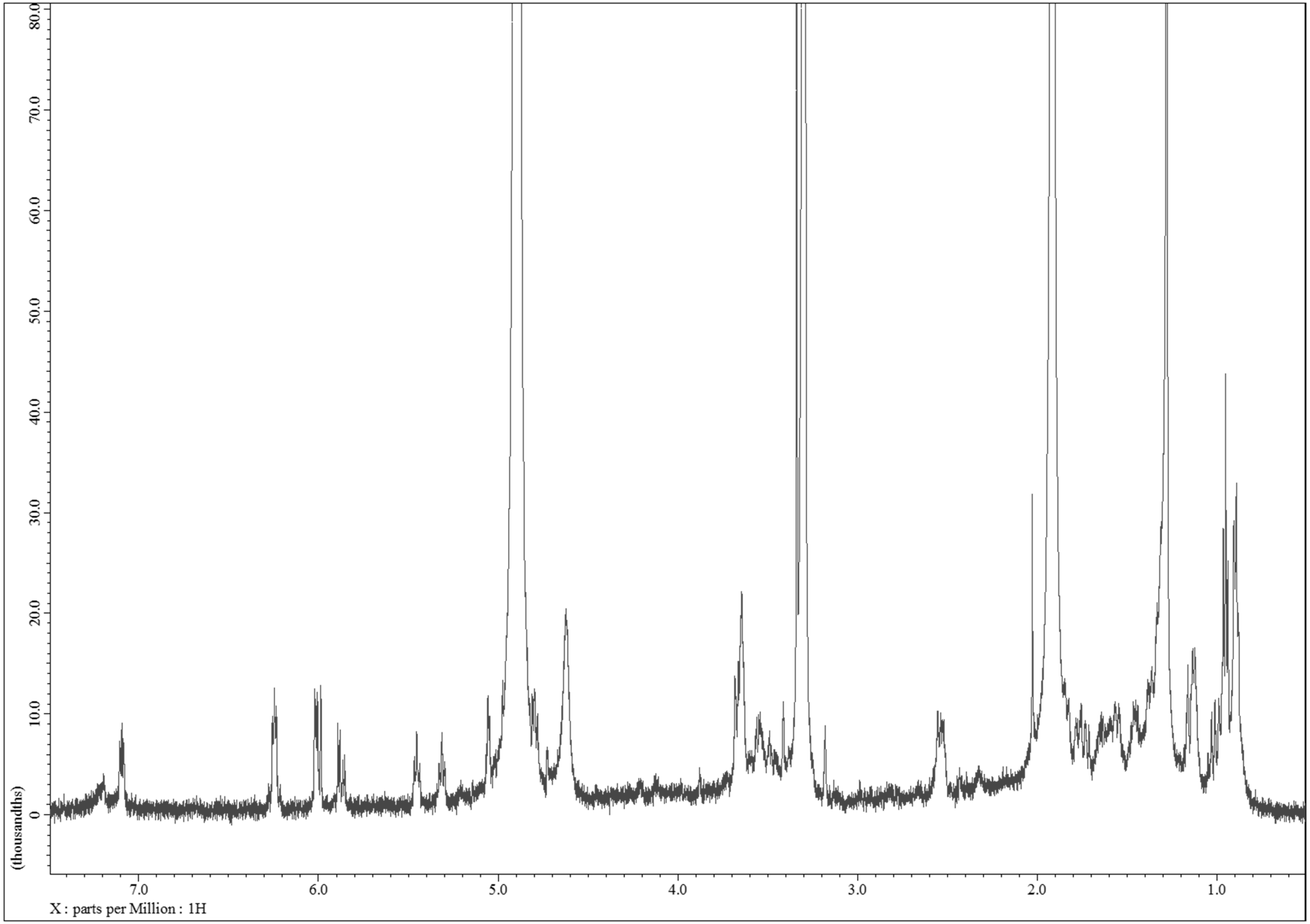

Figure S12. ^{1}H NMR spectrum of lactomycin C (**3**) in CD_3OD.

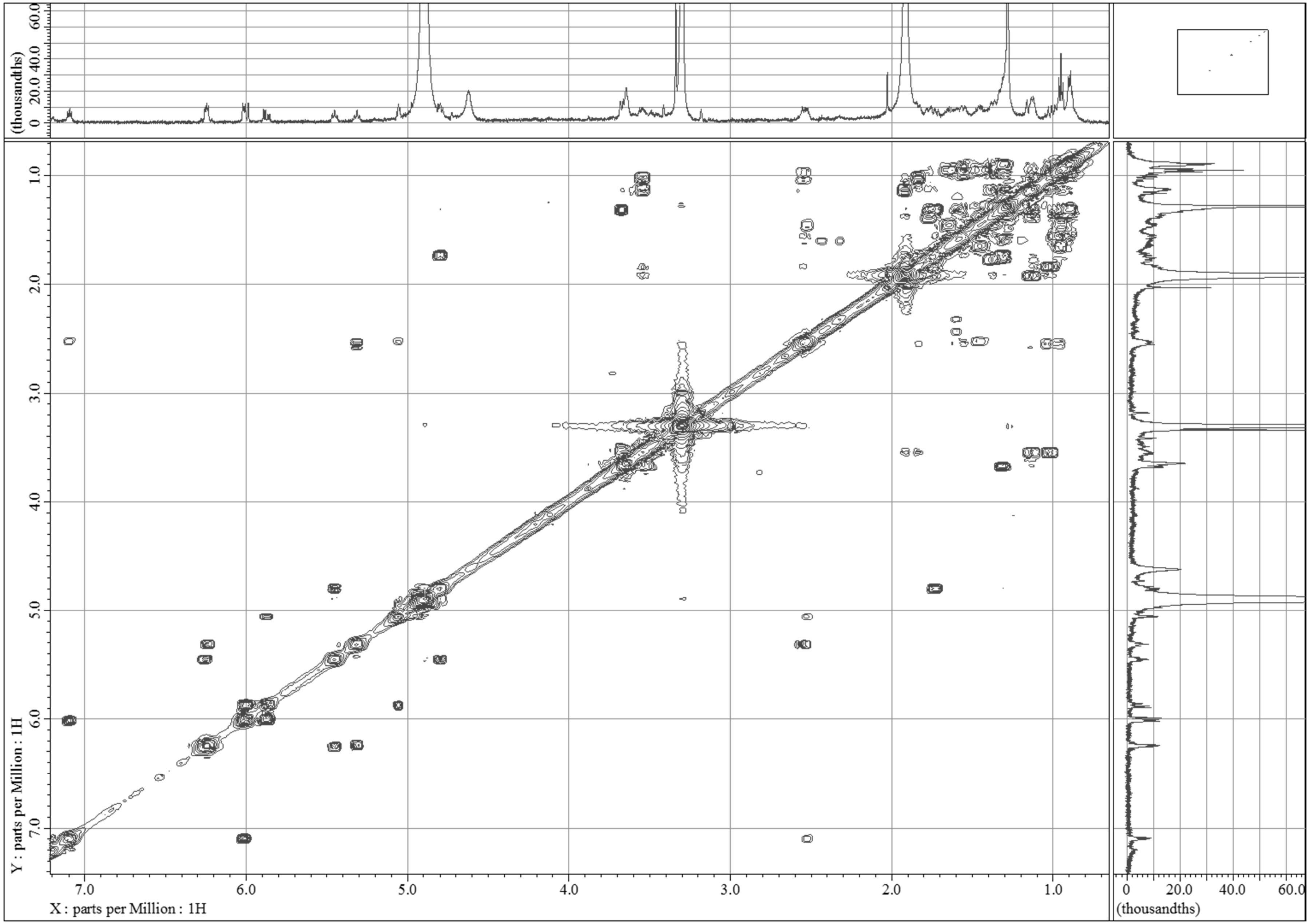

Figure S13. COSY spectrum of lactomycin C (**3**) in CD_3OD.

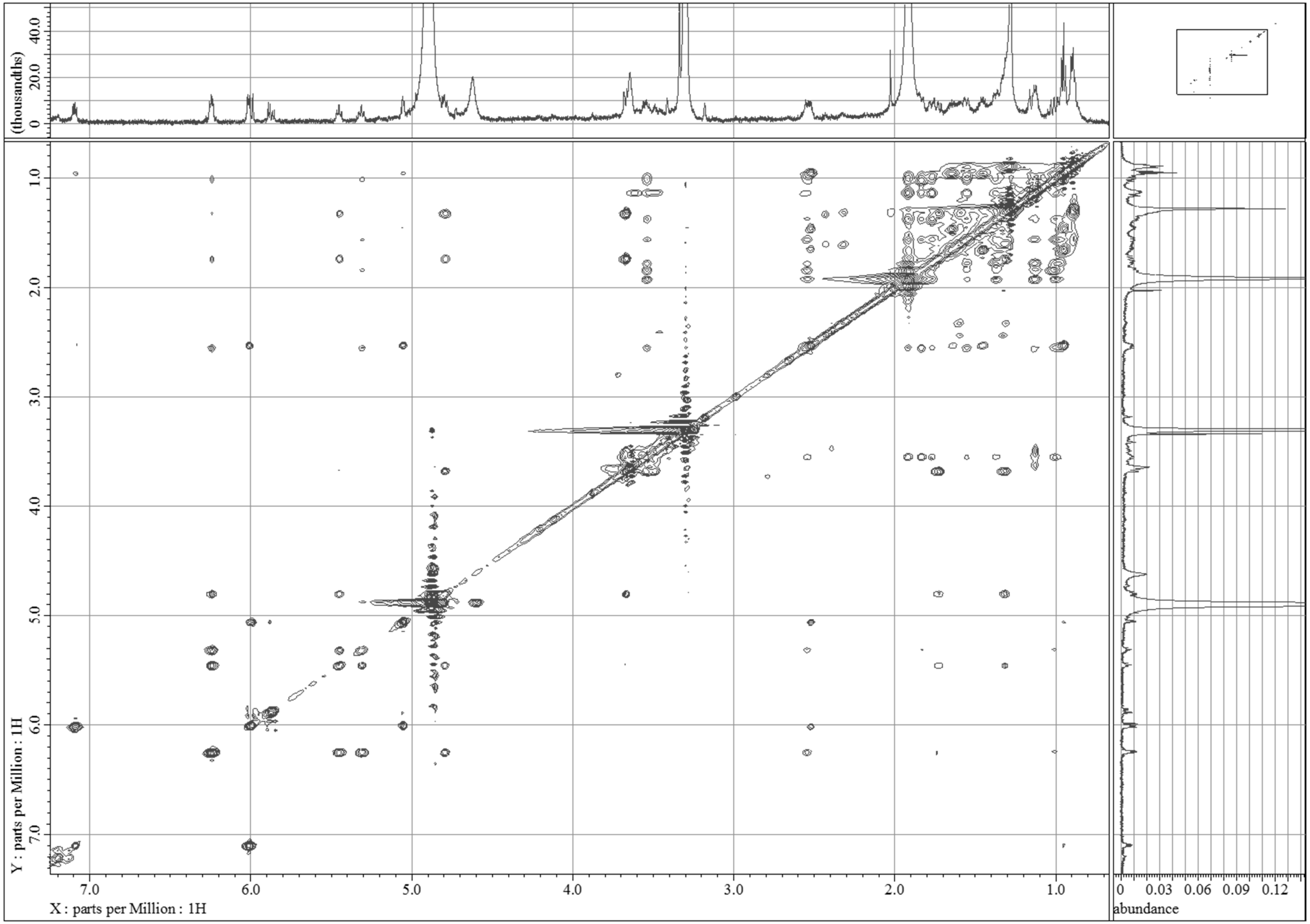

Figure S14. TOCSY spectrum of lactomycin C (**3**) in CD_3OD.

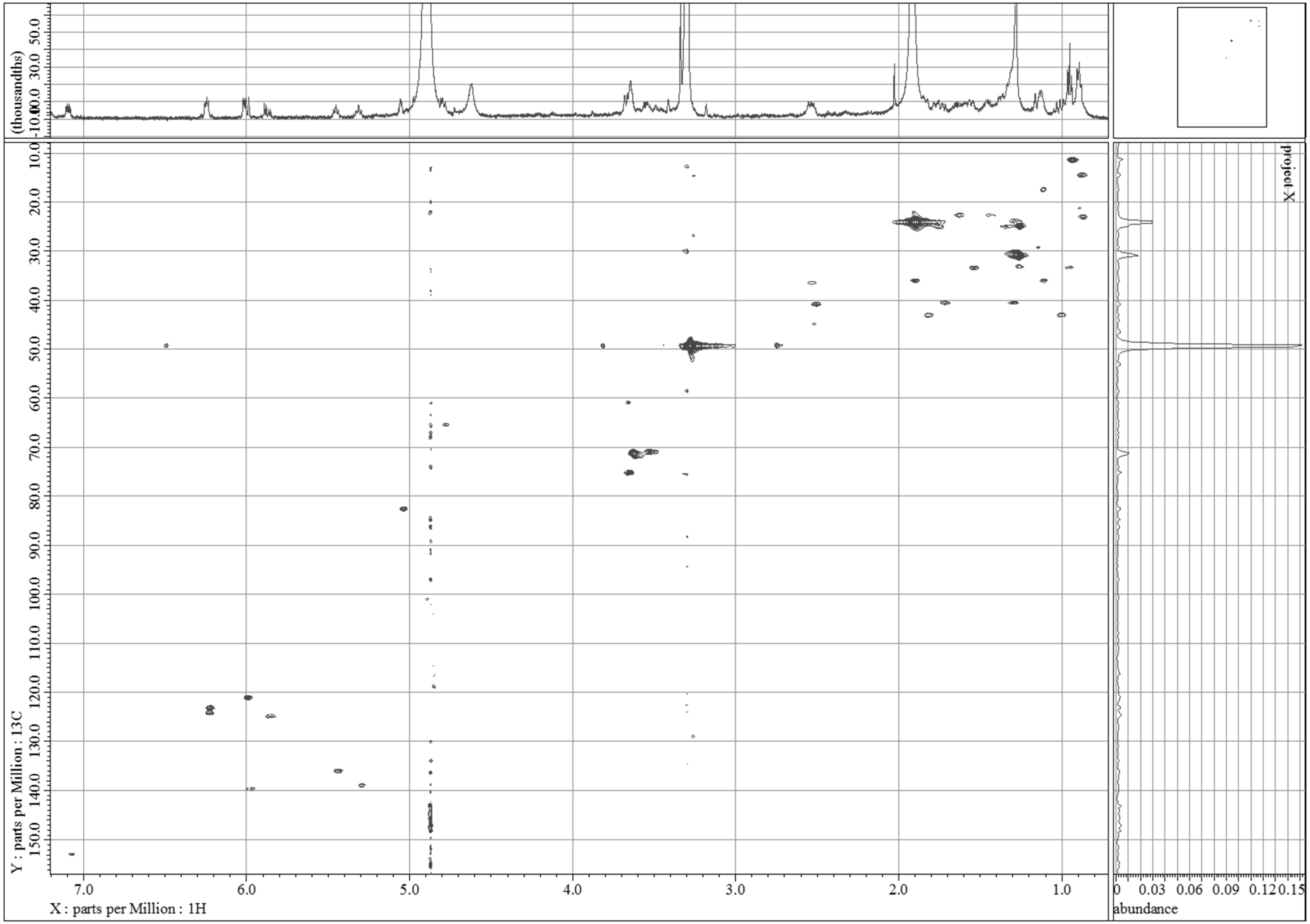

Figure S15. HSQC spectrum of lactomycin C (**3**) in CD_3OD.

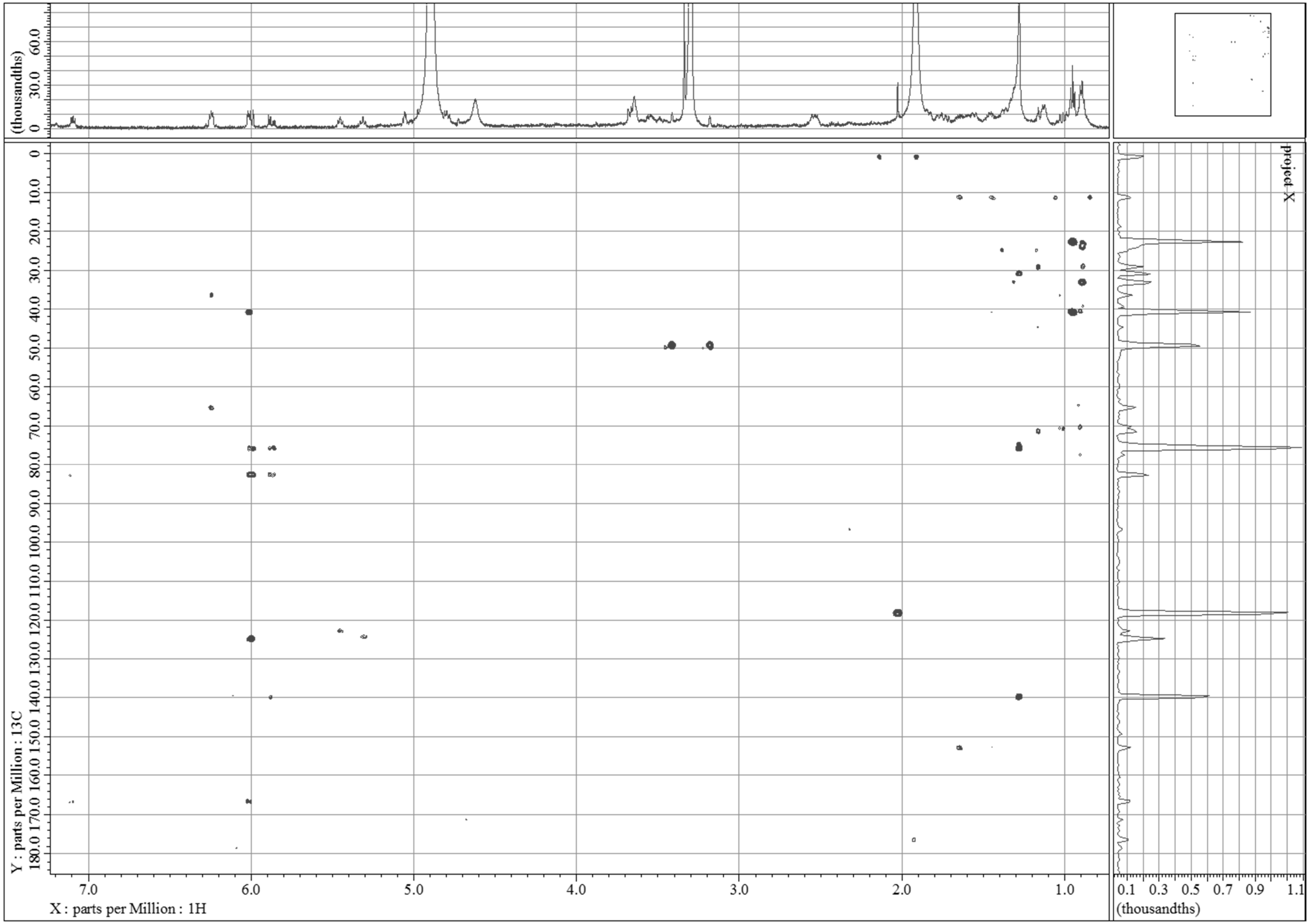

Figure S16. HMBC spectrum of lactomycin C (**3**) in CD_3OD.
